# Prevalence and significance of clonal hematopoiesis of indeterminate potential in lung transplant recipients

**DOI:** 10.1186/s12890-023-02703-1

**Published:** 2023-10-30

**Authors:** Aparna C. Swaminathan, Richard Barfield, Mengqi Zhang, Gundula Povysil, Cliburn Chen, Courtney Frankel, Francine Kelly, Matthew McKinney, Jamie L. Todd, Andrew Allen, Scott M. Palmer

**Affiliations:** 1 Duke Clinical Research Institute, Durham, NC USA; 2 Department of Medicine, Duke University Medical Center, Durham, NC USA; 3 Department of Biostatistics and Bioinformatics, Duke University Medical Center, Durham, USA; 4 Center for Human Systems Immunology, School of Medicine, Duke University, Durham, USA; 5 Institute for Genomic Medicine, Columbia University Medical Center, New York, NY USA

## Abstract

**Background:**

Clonal hematopoiesis of indeterminate potential (CHIP), the age-related acquisition of somatic mutations that leads to an expanded blood cell clone, has been associated with development of a pro-inflammatory state. An enhanced or dysregulated inflammatory response may contribute to rejection after lung transplantation, however the prevalence of CHIP in lung recipients and influence of CHIP on allograft outcomes is unknown.

**Methods:**

We analyzed whole-exome sequencing data in 279 lung recipients to detect CHIP, defined by pre-specified somatic mutations in 74 genes known to promote clonal expansion of hematopoietic stem cells. We compared the burden of acute rejection (AR) over the first post-transplant year in lung recipients with vs. without CHIP using multivariable ordinal regression. Multivariate Cox proportional hazards models were used to assess the association between CHIP and CLAD-free survival. An exploratory analysis evaluated the association between the number of CHIP-associated variants and chronic lung allograft dysfunction (CLAD)-free survival.

**Results:**

We detected 64 CHIP-associated mutations in 45 individuals (15.7%), most commonly in *TET2* (10.8%), *DNMT3A* (9.2%), and *U2AF1* (9.2%). Patients with CHIP tended to be older but did not significantly differ from patients without CHIP in terms of race or native lung disease. Patients with CHIP did not have a higher incidence of AR over the first post-transplant year (p = 0.45) or a significantly increased risk of death or CLAD (adjusted HR 1.25, 95% CI 0.88–1.78). We did observe a significant association between the number of CHIP variants and CLAD-free survival, specifically patients with 2 or more CHIP-associated variants had an increased risk for death or CLAD (adjusted HR 3.79, 95% CI 1.98–7.27).

**Conclusions:**

Lung recipients have a higher prevalence of CHIP and a larger variety of genes with CHIP-associated mutations compared with previous reports for the general population. CHIP did not increase the risk of AR, CLAD, or death in lung recipients.

**Supplementary Information:**

The online version contains supplementary material available at 10.1186/s12890-023-02703-1.

## Introduction

Lung transplantation is an established therapy for many end-stage lung diseases; however, outcomes remain suboptimal, with a median post-transplant survival of about 6.5 years [1]. The primary barrier to better long-term outcomes after lung transplant is chronic lung allograft dysfunction (CLAD), which accounts for death in 40 to 50% of recipients after the first post-transplant year [2]. The pathogenesis of CLAD is complex and not well understood, though may involve dysregulation of the immune response preventing normal wound healing in the lung. Supporting this, germline genetic polymorphisms that alter the expression or function of innate immune receptors influence the risk of CLAD development, likely due to differences in innate immune activation [3, 4].

An increase in immune activation has recently been noted in individuals with clonal hematopoiesis of indeterminate potential (CHIP) [5], a phenomenon in which hematopoietic stem cells acquire somatic mutations in leukemogenic driver genes in an individual without other hematologic abnormalities [6, 7]. The prevalence of CHIP increases with age, affecting 10 to 20% of people age 70 to 80 years. Other risk factors for CHIP include tobacco use and germline variants in telomere-associated genes such as *TERT* [8–10]. Mutations in the genes most frequently identified in CHIP, Tet methylcytosine dioxygenase 2 (*TET2*) and DNA methyltransferase 3 alpha (*DNMT3A)*, have been linked to an increase in pro-inflammatory cytokine/chemokine production and altered immune cell function [6, 11–15].

CHIP is associated with a broad range of health consequences in asymptomatic individuals, including early death due an increased incidence of atherosclerotic conditions [16, 17] and an increased susceptibility to severe infections [18, 19]. However, the prevalence of CHIP and its impact on CLAD development after lung transplantation is not well established. This is particularly relevant given the increasing trend towards transplanting older recipients, in whom the prevalence and potential implications of CHIP may be greater [20]. Therefore, to better understand the frequency of CHIP in lung transplant recipients and the association of CHIP with post-transplant outcomes, we analyzed a single center cohort precisely genotyped through whole-exome analysis. We hypothesized that patients with CHIP would experience higher rates of acute rejection (AR) and worse CLAD-free survival.

## Methods

### Study Population

The study cohort was comprised of 280 adult, first lung recipients primarily with pulmonary fibrosis at Duke University Medical Center previously consented for DNA collection. All patients underwent whole sequencing, and some were included in a prior study focused on genetics of pulmonary fibrosis [21]. One subject was excluded due to concern of whole exome sequencing contamination, resulting in a final analysis cohort of 279 patients. Given lung transplant requirements, there were no patients with active malignancies. Clinical management of patients after lung transplant at Duke has been described previously [22]. Briefly, patients received induction immunosuppression with basiliximab and maintenance immunosuppression with a calcineurin inhibitor (tacrolimus or cyclosporine), cell-cycle inhibitor (mycophenolate mofetil or azathioprine), and prednisone. All recipients underwent pulmonary function testing and transbronchial lung biopsies after transplant as previously described [23]. Clinical information was retrospectively extracted from the electronic medical record. This study was approved through the Duke University institutional review board (Duke IRB Protocol 00100758).

### CHIP identification

All subjects previously underwent exome sequencing of blood-extracted DNA using the NimbleGen SeqCap EZ version 2.0 or 3.0 exome enrichment kit (Roche NimbleGen, Madison, WI) on HiSeq 2000 or 2500 sequencers (Illumina, San Diego, CA) according to standard protocols [21]. Whole exome sequencing data was evaluated for CHIP using the GATK MuTECT2 somatic variant caller [24, 25]. The approach to CHIP identification was consistent with prior studies [6, 10] and based on a pre-specified list of leukemogenic driver mutations in 74 genes known to promote clonal expansion of hematopoietic stem cells. All variants with a variant allele frequency (VAF) of at least 0.02 (2%) were considered according to previously established definitions [26].

To identify subjects with CHIP, we first removed germline variants using germline filters on MuTECT2. We also removed variants with VAF greater than 50%, as these were more likely to be germline variants. We used the Ensembl Variant Effect Predictor [27] to exclude synonymous variants and limit identified variants to those predicted to be of high or moderate impact. We applied the consensus coding sequence (CCDS) region list (with a pad of 10 base pairs) to ensure all identified variants are located in exons.

To ensure sequence quality, we applied the FilterMutectCalls from GATK pipeline to remove estimated contamination. In addition, we required each variant to have a read depth of at least 10 base pairs and at least one read from each DNA strand aligned to the variant position. To estimate sequencing artifacts, we evaluated the number of variants in 12,154 genes not expressed by peripheral blood cells as well as the number of somatic variants in the entire exome. One subject had a very high number of somatic single nucleotide variants in both exome and non-blood expressed genes, and thus the subject was removed from the study cohort.

### Study outcomes

The study outcomes were (1) burden of AR over the first post-transplant year, and (2) CLAD-free survival. AR was defined as perivascular infiltrates detected on transbronchial biopsies and graded according to severity (A0-A4) as described by International Society for Heart and Lung Transplant (ISHLT) guidelines [28]. Episodes of AR were considered distinct if they occurred at least six weeks apart. An AR score was calculated for the first year after transplant, defined as the sum of ISHLT grade A scores divided by the total number of transbronchial lung biopsies with gradable rejection scores [29, 30].

Survival was defined as graft survival and was calculated from date of transplant to either death, re-transplant, or last known date of active clinical follow up. Consistent with the international definition, CLAD was defined as a persistent decline in forced expiratory volume in 1 s (FEV1) less than 80% of baseline (average of 2 best FEV1 values after transplantation) in the absence of other confounding conditions [31]. In order to be eligible for CLAD assessment, recipients must have survived at least 90 days and undergone at least five pulmonary function tests post-transplant, consistent with the approach in prior publications [30, 32].

### Statistical analysis

For descriptive statistics, we present medians with 25th and 75th percentiles for continuous variables and frequencies with percentages for categorical variables. To evaluate associations between CHIP and baseline clinical/demographic characteristics as well as mean sequencing depth, we used t-tests and fisher’s-exact tests for continuous and categorical variables respectively. The Kaplan Meier method was used to obtain estimates of the cumulative event probability as a function of time by the presence of CHIP, as well as the number of CHIP variants (0, 1, or ≥ 2) for the outcome of CLAD-free survival.

To test the hypothesis that patients with CHIP would experience higher burden of AR over the first post-transplant year, we first assessed the association between median AR score and CHIP variants via a Wilcoxon Rank Sum test. We then fit a multivariable ordinal regression mixed effects model for the probability of an A1 or an A2 and higher AR episode in patients with CHIP compared to those without CHIP. The model was adjusted for age at transplant, sex, transplant type, and total number of human leukocyte antigen (HLA) mismatches between donor and recipient (0–3 vs. 4–6) as determined by HLA genotyping. An exploratory analysis was performed to determine if the number of CHIP variants (0, 1, or ≥ 2) was associated with AR using the multivariable ordinal regression model.

To test the hypothesis that patients with CHIP had an increased risk for CLAD or death, we used a multivariable Cox proportional hazards model for time-to-first event as a function of the binary indicator of presence vs. absence of CHIP. Covariates in our model were: type of transplant (single vs. bilateral), age at transplant, sex, and grade 3 primary graft dysfunction (PGD) at 48 or 72 h, defined according to ISHLT consensus criteria [33]. The proportional hazards assumption was checked using the time-interaction test and was found to be violated for the transplant type variable. Since CLAD-free survival differed over the first 1,200 days for single lung transplants versus bilateral lung transplants, we stratified the analysis by transplant type over that time period.

To further evaluate the association of CHIP with CLAD we performed additional pre-specified exploratory analyses. First, we considered if the number of CHIP-associated variants was associated with CLAD-free survival using a multivariable Cox proportional hazards model with the above covariates. CHIP was coded as either no CHIP, 1 CHIP-associated variant, or ≥ 2 CHIP-associated variants. Second, we compared CLAD-free survival among individuals with a CHIP-associated variant in *TET2* or *DNMT3A* versus other leukemogenic driver genes or no CHIP variant, given that prior studies in cardiovascular disease suggested adverse events are driven by mutations in *TET2* and *DNMT3A* [34, 35]. Finally, given previous observations of an association between rare, telomere-related gene variants and both CLAD and death [22], we included the presence of a qualifying telomere-related gene variant as an adjustment covariate in the association of CHIP with CLAD-free survival. We also performed an interaction analysis to determine if the association between CHIP and CLAD-free survival varied based on the presence of a qualifying telomere-related gene variant. The presence of qualifying variants in telomere-related genes in patients with pulmonary fibrosis was previously assessed using an exome-wide rare variant collapsing analysis [21]. All qualifying variants were protein coding variants that met specific criteria including a rare population allele frequency and predicted *in silico* damaging effect [21].

## Results

### Cohort characteristics and prevalence of CHIP

**Fig. 1:**
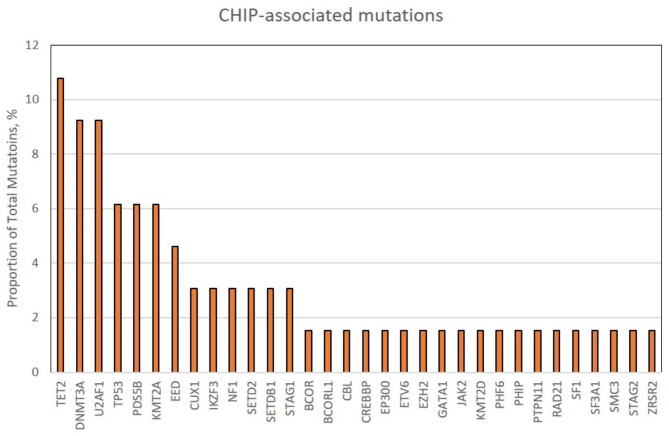
Proportion of CHIP-associated mutations by leukemogenic driver gene

The 279 adult, first lung recipient study cohort was predominantly male (66.7%) with a median age at transplant of 60 (Table 1). The most common disease indication for transplant was pulmonary fibrosis (204/279, 73.1%). CHIP-associated variants were detected in 45 individuals (45/279, 15.7%) who carried a total of 64 mutations in 32 genes with a median (Q1,Q3) VAF of 6.2% (4.6–9.2). Patients with CHIP tended to be older (Supplemental Fig. 1) but did not significantly differ from patients without CHIP in terms of race or native lung disease (Table 1). The mean sequencing depth was similar among individuals with CHIP compared to those without (82.81 vs. 77.55, p = 0.12, Supplemental Fig. 2). Among patients with CHIP, 35 (35/45, 78%) had a mutation in only one CHIP-associated gene, and 10 individuals (10/45, 22%) had mutations in 2 or more CHIP-associated genes (Supplemental Table 1). A list of all identified CHIP-associated mutations is included in Supplemental Table 2. The most frequently mutated genes were *TET2* (10.8%), *DNMT3A* (9.2%), and *U2AF1* (9.2%, Fig. 1).

**Table 1:** Demographic and Clinical Characteristics of the Study Cohort, overall and stratified by presence or absence of CHIP

	Entire Cohort N = 279	CHIP present N = 45	CHIP absent N = 234	*p-value* ^*a*^
**Transplant Age, years**	58.3 (11.4)	60.9 (10.6)	57.8 (11.5)	0.08
**Male**	186 (66.7%)	26 (57.8%)	160 (68.4%)	0.17
**Race**				0.46
European	250 (89.6%)	42 (93.3%)	208 (88.9%)	
Black	24 (8.6%)	2 (4.4%)	22 (9.4%)	
Asian	3 (1.1%)	1 (2.2%)	2 (0.9%)	
**Ethnicity: Hispanic**	3 (1.1%)	1 (2.2%)	2 (0.9%)	0.41
**UNOS native disease**				0.81
Obstructive	44 (15.8%)	8 (17.8%)	36 (15.4%)	
Vascular	4 (1.4%)	1 (2.2%)	3 (1.3%)	
Cystic	17 (6.1%)	2 (4.4%)	15 (6.4%)	
Restrictive	214 (76.7%)	34 (75.6%)	180 (76.9%)	
**Bilateral Lung Transplant**	210 (75.3%)	33 (73.3%)	177 (75.6%)	0.71
**CMV serological status mismatch (D + R-)**	54 (19.8%)	12 (27.3%)	42 (18.3%)	0.24
**Assessed for telomere-related gene variant** ^b^	173 (62.0%)	29 (64.4%)	144 (61.5%)	0.74
**Telomere-related gene variant present** ^b^	23/173 (13.2%)	7/29 (24.1%)	16/144 (11.1%)	0.06

Continuous variables displayed as mean (SD); Categorical variables displayed as n (%). ^a^ Evaluated with t-tests and fisher’s-exact tests for continuous and categorical variables respectively.

^b^ Evaluated in the subset of patients with pulmonary fibrosis as previously described (21)

A subset of patients with pulmonary fibrosis (173/204) were previously evaluated for qualifying variants in telomere-related genes in *TERT, RTEL1*, or *PARN* [21]. Given prior associations of CHIP with germline *TERT* variants [10], we considered whether CHIP was more frequent in patients with qualifying telomere-related gene variants. While not statistically significant, patients with pulmonary fibrosis and CHIP were numerically more likely to have a qualifying telomere-related gene variant present compared to pulmonary fibrosis patients without CHIP (7/29, 24% vs. 16/144, 11%, p = 0.06, Table 1).

### CHIP and acute rejection

A total of 269 patients had at least one transbronchial lung biopsy in the first year after transplant and were thus included in the AR analyses. In the first post-transplant year, the proportion of patients who experienced an episode of AR graded A1 or higher was similar among patients with CHIP compared to those without CHIP (57% vs. 52%, Supplemental Table 3). There was also no significant difference in the median AR score (reflecting both the frequency and severity of rejection) in patients with CHIP compared to those without CHIP variant (0.32 vs. 0.33, p = 0.17, Supplemental Fig. 3). In a multivariable ordinal regression model with a random intercept for patient, there was no statistically significant association between CHIP and AR (p = 0.45, Table 2). There was also no association between the presence of ≥ 2 CHIP-associated variants vs. no CHIP and AR (Table 2).

**Table 2:** Association between CHIP and AR in the first post-transplant year a

*Variable*	*Log Odds Estimate*	*p-value*
CHIP ^b^	0.14	0.45
Covariates ^c^		
Age at transplant ^d^	0.00265	0.68
Female	0.003	0.88
Single lung transplant	-0.30	0.07
HLA mismatch between 4–6	-0.105	0.55

^a^ Evaluated by fitting an ordinal regression mixed effects model

^b^ There was no association between number of CHIP variants and AR (p = 0.45)

^c^ Intercepts: A0|A1(1.27); A1|A2 or higher (2.32)

^d^ Mean centered

### CHIP and CLAD-free survival

Over a median follow up of 3.49 years, 83.5% of the cohort (233/279 individuals) either developed CLAD, required re-transplant, or died. Specifically, 21 of 45 patients with CHIP (46.7%) developed CLAD compared with 98/234 (41.9%) of patients without CHIP (Supplemental Table 4). The cause of death for most patients with CHIP (26/33, 78.8%) was graft failure.

**Fig. 2:**
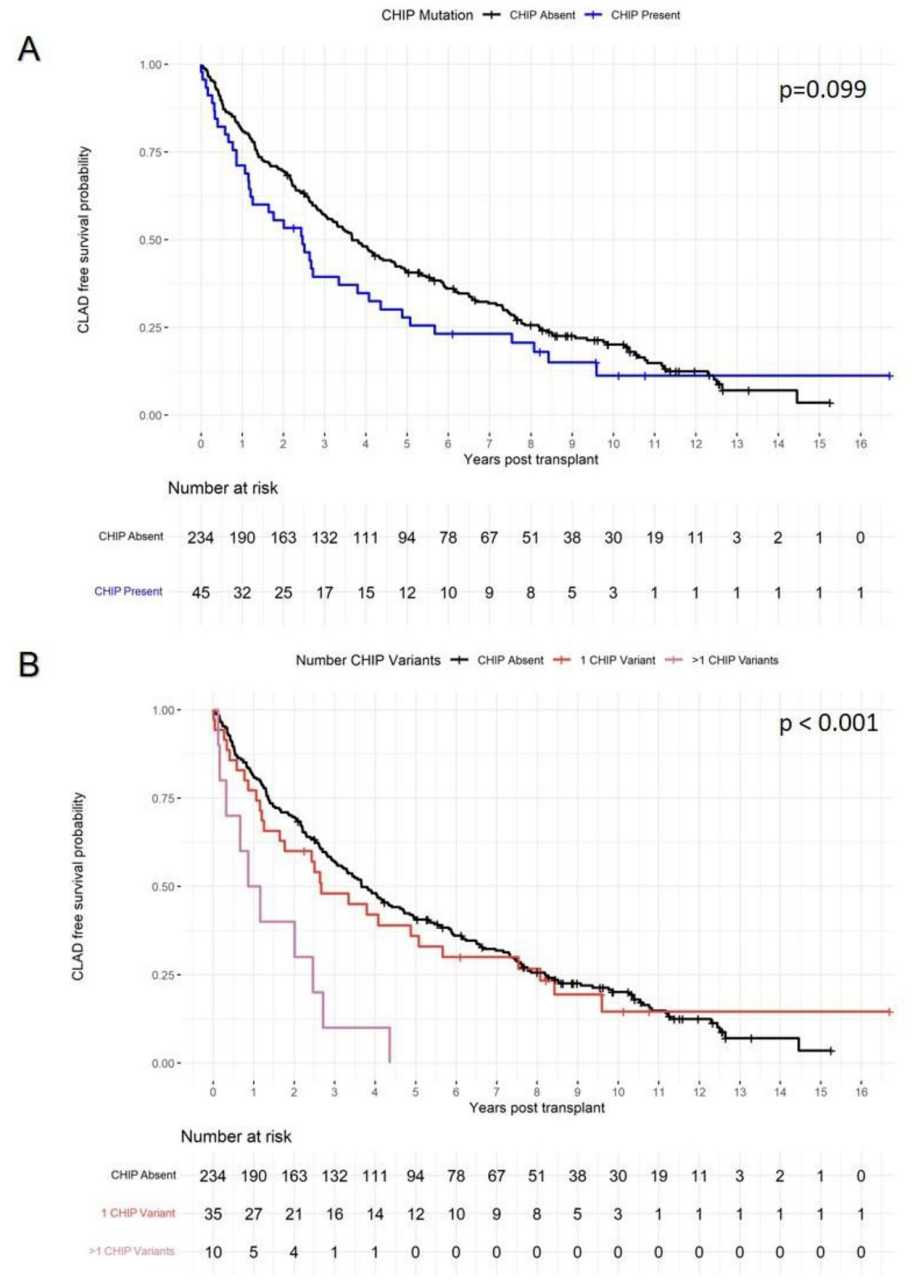
Kaplan Meier curve with p-values from the log rank test for time to development of CLAD or death after lung transplant stratified by (A) presence of CHIP and (B) number of CHIP-associated variants

Kaplan Meier estimates at one, three, and five years for CLAD-free survival were lower in patients with CHIP compared to those without CHIP (71.1%, 39.4%, and 27.8% versus 81.2%, 57.2%, and 41.1%, Fig. 2A). However, patients with CHIP did not have a statistically significant increased risk of CLAD or death in univariable (HR 1.34, 95% CI 0.95–1.90, p = 0.10) or multivariable (HR = 1.25, 95% CI: 0.88–1.78, p = 0.21) analyses. In an exploratory analysis that considered number of CHIP variants, we observed an overall difference in CLAD-free survival by number of CHIP variants. Patients with 2 or more CHIP variants had an increased risk of CLAD or death compared to patients without CHIP variants in both univariable (HR 3.49, 95% CI 1.83–6.67, p < 0.001) and multivariable (HR 3.79, 95% CI 1.98–7.27, p < 0.001) analyses (Table 3; Fig. 2B).

**Table 3:** Association between CHIP and CLAD or death a

*Variable*	*HR (95% CI)*	*p-value*
CHIP-associated variant		0.0003
1 CHIP-associated variant	1.00 (0.67–1.50)	
≥ 2 CHIP-associated variants	3.79 (1.98–7.27)	
Age at transplant	1.02 (1.01–1.03)	0.008
Female	1.1 (0.83–1.46)	0.49
PGD ^b^	1.59 (1.08–2.34)	0.02
Single lung transplant ^c^		
Before 1200 days	0.86 (0.56–1.31)	0.48
After 1200 days	2.40 (1.44–3.99)	0.0007

CHIP = clonal hematopoiesis of indeterminate potential, CLAD = chronic lung allograft dysfunction, PGD = primary graft dysfunction

^a^ Evaluated using multivariable Cox proportional hazards model

^b^ Grade 3 at 48 or 72 h post-transplant

^c^ Stratified due to originally violating the proportional hazards assumption.

We next considered CHIP-associated mutations only in *TET2* or *DNMT3A.* Among the 13 patients with a CHIP-associated variant in *TET2* or *DNMT3A*, 30.8% (4/13) had more than one CHIP-associated variant present, while the remainder had only one CHIP-associated variant present. There was no statistically significant difference in CLAD-free survival among patients with a *TET2* or *DNMT3A* variant compared to subjects without CHIP (HR 1.28, 95% CI 0.68–2.4, p = 0.44, Supplemental Table 5).

Given the previously observed association of qualifying telomere-related gene variants with CLAD and death in patients with pulmonary fibrosis, we performed an exploratory analysis in the subset of patients with pulmonary fibrosis that also adjusted for telomere-related gene variants. Among 173 patients with pulmonary fibrosis, the presence of more than one CHIP variant associated with an increased risk for death or CLAD after adjustment for qualifying telomere-related gene variants (HR 4.08, 95% CI 1.95–8.55, Supplemental Table 6). Additionally, in a model that considered telomere-related variants as an interacting term with the presence or absence of CHIP, we did not observe a significant interaction between CHIP and telomere-related gene variants (Supplemental Figs. 4&5).

## Discussion

Clonal hematopoiesis has been associated with an increased inflammatory state as well as increased incidence of and mortality from atherosclerotic conditions [16]. However, the prevalence and significance of CHIP in solid organ transplantation is not well described. We evaluated a cohort of lung recipients and observed a higher prevalence of CHIP compared with previously published data in age-matched healthy participants as well as an increase in the number of genes with CHIP-associated mutations [7]. We hypothesized that patients with CHIP would have worse CLAD-free survival, but did not identify a significant association between CHIP and CLAD or death. We did observe an overall difference in CLAD-free survival by number of CHIP-associated variants. Specifically, patients with two or more CHIP-associated variants had an increased risk of death or CLAD compared to patients without CHIP.

To our knowledge, this is the first study to examine the impact of CHIP on allograft rejection in lung recipients. A recent analysis of 85 lung recipients identified an association between clonal hematopoiesis in DNA damage response genes and an increased risk of cytomegalovirus activation and mycophenolate intolerance [36], both of which could increase risk of CLAD development. Further, prior studies evaluating CLAD pathogenesis have suggested a role for IL-1β and IL-6 [37, 38], cytokines which can also be elevated in the peripheral blood of individuals with CHIP [39], leading to our hypothesis that CHIP increases the risk of CLAD development. However most prior studies did not evaluate patients treated with immunosuppression, which could diminish proinflammatory cytokine expression and attenuate the effect of CHIP. In fact, blockade of targets such as IL-1β and IL-6 has been proposed as a potential strategy to mitigate the effects of CHIP [40]. Thus, one reason an association between CHIP and an increased risk of CLAD or death was observed only in patients with two or more CHIP-associated mutations could be because these patients had a higher burden of CHIP despite post-transplant immunosuppression.

Another potential reason that an association between CHIP and CLAD-free survival was not observed in all patients is the increase in distribution of CHIP-associated genes in our cohort compared to prior studies [6, 9, 35]. While the most commonly mutated genes in lung recipients with CHIP were the epigenetic regulators *DNMT3A* and *TET2*, the proportion of mutations in *DNMT3A* (11%) and *TET2* (9%) in our cohort was much lower than prior studies of patients with heart failure [41], coronary heart disease [6], COPD [42], and healthy volunteers [7], where about 70% of variants were in *DNMT3A* or *TET2*. Studies in cardiovascular disease suggest adverse events associated with CHIP are driven by *TET2* and *DNMT3A* [34, 35]. We separately analyzed CLAD-free survival in the 13 participants with CHIP-associated variants in *TET2* or *DNMT3A*, but did not identify a statistically significant association.

The prevalence of CHIP increases with aging, and early studies identified CHIP in over 10% of individuals older than 70 years of age [7]. In the current study, CHIP was identified in 16% of lung recipients despite the younger age range. This is consistent with recent work that observed a 20% prevalence of clonal hematopoiesis in heart transplant recipients [43], but lower than an evaluation of 73 lung recipients that identified CHIP in about 70% of the cohort using an error-corrected sequencing assay able to detect a VAF as low as 0.01 [44]. Similar highly sensitive sequencing assays have also revealed a greatly increased prevalence of clonal hematopoiesis in healthy older adults [45]. The threshold of clonal expansion that is clinically meaningful with regards to adverse outcome prognostication remains an area of active investigation.

With regards to indications for lung transplant, the prevalence of CHIP has been best evaluated in individuals with chronic obstructive pulmonary disease (COPD), where it was identified in 6% of patients with COPD of a similar age range as our cohort [42]. The prevalence of CHIP in our cohort did not differ by lung transplant indication. However, among patients with pulmonary fibrosis, we observed a higher prevalence of CHIP in patients who carried a qualifying variant in a telomere-related gene compared to non-carriers, which differs from a recent study [36], possibly related to our larger sample size and focus on rare variants. Both pulmonary fibrosis and CHIP are conditions related to aging, and both are also associated with germline variants in *TERT* [8, 10, 21, 46, 47]. In fact, a recent case series of patients with germline variants in telomere-maintenance genes found a 30% prevalence of CHIP [48] and recent mendelian randomization analysis suggests that CHIP may cause telomere shortening [49]. Given the association of both CHIP and pulmonary fibrosis with impaired telomere maintenance, future studies should evaluate the role of CHIP in development and progression of pulmonary fibrosis.

We did not observe an association between CHIP and AR over the first post-transplant year. In fact, very few factors were associated with AR in our cohort, though the direction of the association for single lung transplant receipt with AR is consistent with a recent multi-center analysis [50]. AR is characterized by an alloreactive T cell response to donor-derived antigens, and T helper (Th) differentiation into Th1 cells that produce interferon gamma and IL-2, which is a different profile of cytokines than has been previously associated with CHIP. A recent evaluation of heart transplant recipients also did not identify an association between CHIP and rejection [43].

There are several limitations to this study. First, the whole exome sequencing data used in this study did not perform target enrichment in the known CHIP genes, and it is thus possible that CHIP variants with a low VAF were not identified. For example, mutations in *ASXL1* were absent in our cohort which contrasts with prior studies and may indicate low coverage of that gene [39]. Second, this was a retrospective, single center, cohort study that may be underpowered to detect associations between CHIP and AR or CLAD. Larger cohorts could also evaluate clinical outcomes such infection or malignancy, and better characterize the influence of immunosuppressive agent. Third, the timing of CHIP ascertainment varied among patients, as it was not always directly before or after transplant, and also was not precisely known for the cohort. While, the development of CHIP is generally thought to occur over decades, it is possible the transplant surgery, post-operative events, or immunosuppression regimens may have influenced development of CHIP mutations. Finally, patients with pulmonary fibrosis were over-represented in this cohort, and future analyses of CHIP in lung transplant recipients should evaluate a cohort with an observed distribution of underlying diseases similar to international registries.

## Conclusions

Our study evaluated the prevalence of a CHIP in lung recipients and its association with AR and CLAD-free survival. Compared with previous reports in healthy individuals, lung recipients have a higher prevalence of CHIP and a larger variety of genes with CHIP-associated mutations. CHIP did not increase the risk of AR, CLAD, or death in lung recipients. high burden of age-related clonal hematopoiesis may be. Further evaluation of CHIPin a larger cohort, particularly patients with a high burden of age-related clonal hematopoiesis, may allow better evaluation of clinical relevance of CHIP in lung transplant recipients.

### Electronic supplementary material

Below is the link to the electronic supplementary material.

Supplementary Material 1

## Data Availability

The data that support the findings of this study are available on request from the corresponding author. The data are not publicly available due to privacy or ethical restrictions.

## Abbreviations

AR: acute rejection
CHIP: clonal hematopoiesis of indeterminate potential
CLAD: chronic lung allograft dysfunction
PGD: primary graft dysfunction
VAF: variant allele frequency
