# Supplementary material for: Prevalence and significance of clonal hematopoiesis of indeterminate potential in lung transplant recipients
Source: BMC Pulm Med. 2023 Oct 30;23:414. doi: 10.1186/s12890-023-02703-1 (PMC10614406; doi:10.1186/s12890-023-02703-1)
Supplement: Supplementary file 1 — Supplementary Material 1 [file 12890_2023_2703_MOESM1_ESM.docx]

**SUPPLEMENTAL MATERIALS**

| **Supplemental Table 1: Demographic and Clinical Characteristics of the Study Cohort, overall and stratified by number of CHIP-associated variants** | | | | | |
| --- | --- | --- | --- | --- | --- |
|  | Entire Cohort  N = 279 | 1 CHIP variant  N= 35 | > 1 CHIP variant, N = 10 | CHIP absent  N = 234 | *p-value ^a^* |
| **Transplant Age, years** | 58.3 (11.4) | 61.6 (8.9) | 58.4 (15.6) | 57.8 (11.5) | 0.18 |
| **Male** | 186 (66.7%) | 19 (54.3%) | 7 (70.0%) | 160 (68.4%) | 0.27 |
| **Race** |  |  |  |  | 0.62 |
| European | 250 (89.6%) | 32 (91.4%) | 10 (100%) | 208 (88.9%) |  |
| Black | 24 (8.6%) | 2 (5.7%) | 0 (0%) | 22 (9.4%) |  |
| Asian | 3 (1.1%) | 1 (2.9%) | 0 (0%) | 2 (0.9%) |  |
| **Ethnicity: Hispanic** | 3 (1.1%) | 1 (2.9%) | 0 (0%) | 2 (0.9%) | 0.41 |
| **UNOS native disease** |  |  |  |  | 0.72 |
| Obstructive | 44 (15.8%) | 7 (20.0%) | 1 (10.0%) | 36 (15.4%) |  |
| Vascular | 4 (1.4%) | 1 (2.9%) | 0 (0%) | 3 (1.3%) |  |
| Cystic | 17 (6.1%) | 1 (2.9%) | 1 (10.0%) | 15 (6.4%) |  |
| Restrictive | 214 (76.7%) | 26 (74.3%) | 8 (80.0%) | 180 (76.9%) |  |
| **Bilateral Lung Transplant** | 210 (75.3%) | 26 (74.3%) | 7 (70.0%) | 177 (75.6%) | 0.87 |
| **Assessed for telomere-related gene variant** ^b^ | 173 (62.0%) | 21 (60.0%) | 8 (80.0%) | 144 (61.5%) | 0.51 |
| **Telomere-related gene variant present** ^b^ | 23/173 (13.2%) | 6 (28.6%) | 1 (12.5%) | 16/144 (11.1%) | 0.10 |
| Continuous variables displayed as mean (SD); Categorical variables displayed as n (%)  ^a^ Evaluated with t-tests and fisher’s-exact tests for continuous and categorical variables respectively  ^b^ Evaluated in the subset of patients with pulmonary fibrosis as previously described ^1^ | | | | | |

| **Supplemental Table 2: List of variants identified as drivers of clonal hematopoiesis in the study cohort** | | | | | | | | | | | |
| --- | --- | --- | --- | --- | --- | --- | --- | --- | --- | --- | --- |
| **Patient ID** | **HUGO symbol** | **Chrom** | **Position** | **Ref allele** | **Alt allele** | **VAF** | **Variant Type** | **Ref Seq** | **DNA change** | **Ref Seq** | **Protein change** |
| 1 | DNMT3A | 2 | 25470962 | A | AC | 0.196 | Frameshift | NM_022552.4 | c.798dup | NP_072046.2 | p.Ser267ValfsTer14 |
| 2 | ZRSR2 | X | 15833978 | G | T | 0.05 | Stop gained | NM_005089.3 | c.736G>T | NP_005080.1 | p.Glu246Ter |
| 3 | TET2 | 4 | 1.06E+08 | G | T | 0.052 | Missense | NM_001127208.2 | c.5818G>T | NP_001120680.1 | p.Val1940Leu |
| 4 | SETDB1 | 1 | 1.51E+08 | G | T | 0.104 | Stop gained | NM_001145415.1 | c.1228G>T | NP_001138887.1 | p.Glu410Ter |
| 4 | TP53 | 17 | 7577538 | C | T | 0.274 | Missense | NM_001126112.2 | c.743G>A | NP_001119584.1 | p.Arg248Gln |
| 5 | STAG2 | X | 1.23E+08 | G | T | 0.039 | Stop gained & splice region | NM_006603.4 | c.817G>T | NP_006594.3 | p.Glu273Ter |
| 6 | PDS5B | 13 | 33225940 | G | T | 0.095 | Splice acceptor | NM_015032.3 | c.109-1G>T |  |  |
| 6 | PHF6 | X | 1.34E+08 | C | A | 0.091 | Stop gained | NM_001015877.1 | c.359C>A | NP_001015877.1: | p.Ser120Ter |
| 7 | PTPN11 | 12 | 1.13E+08 | C | T | 0.064 | Missense | NM_002834.3 | c.215C>T | NP_002825.3 | p.Ala72Val |
| 8 | TP53 | 17 | 7578417 | C | A | 0.133 | Missense | NM_001126112.2 | c.513G>T | NP_001119584.1 | p.Glu171Asp |
| 9 | TET2 | 4 | 1.06E+08 | C | T | 0.166 | Stop gained | NM_001127208.2 | c.3646C>T | NP_001120680.1 | p.Arg1216Ter |
| 10 | EED | 11 | 85988959 | G | T | 0.078 | Splice acceptor | NM_003797.3 | c.1126-1G>T |  |  |
| 11 | DNMT3A | 2 | 25464450 | C | T | 0.19 | Missense | NM_022552.4 | c.2063G>A | NP_072046.2 | p.Arg688His |
| 12 | TET2 | 4 | 1.06E+08 | AC | A | 0.07 | Frameshift | NM_001127208.2 | c.4158del | NP_001120680.1 | p.His1386GlnfsTer62 |
| 13 | DNMT3A | 2 | 25467465 | G | T | 0.103 | Stop gained | NM_022552.4 | c.1611C>A | NP_072046.2 | p.Cys537Ter |
| 13 | KMT2A | 11 | 1.18E+08 | TG | T | 0.03 | Frameshift | NM_005933.3 | c.2764del | NP_005924.2 | p.Ala922ProfsTer27 |
| 13 | STAG1 | 3 | 1.36E+08 | CTA | C | 0.048 | Frameshift and Stop gained | NM_005862.2 | c.3144_3145del | NP_005853.2 | p.Tyr1048Ter |
| 14 | EED | 11 | 85975258 | GC | G | 0.028 | Frameshift | NM_003797.3 | c.680del | NP_003788.2 | p.Ala227GlufsTer6 |
| 14 | TET2 | 4 | 1.06E+08 | C | A | 0.05 | Missense | NM_001127208.2 | c.4396C>A | NP_001120680.1 | p.Gln1466Lys |
| 14 | U2AF1 | 21 | 44524456 | G | A | 0.066 | Missense | NM_006758.2 | c.101C>T | NP_006749.1 | p.Ser34Phe |
| 14 | U2AF1 | 21 | 44524456 | G | T | 0.066 | Missense | NM_006758.2 | c.101C>A | NP_006749.1 | p.Ser34Tyr |
| 15 | NF1 | 17 | 29679398 | GC | G | 0.056 | Frameshift | NM_000267.3 | c.7519del | NP_000258.1 | p.Gln2507AsnfsTer20 |
| 16 | KMT2D | 12 | 49448320 | C | A | 0.077 | Stop gained | NM_003482.3 | c.391G>T | NP_003473.3 | p.Glu131Ter |
| 17 | GATA1 | X | 48650407 | GA | G | 0.052 | Frameshift | NM_002049.3 | c.381del | NP_002040.1 | p.Gly128AlafsTer9 |
| 18 | TET2 | 4 | 1.06E+08 | C | A | 0.033 | Missense | NM_001127208.2 | c.3451C>A | NP_001120680.1 | p.Leu1151Ile |
| 19 | DNMT3A | 2 | 25463308 | G | C | 0.184 | Missense | NM_022552.4 | c.2185C>G | NP_072046.2 | p.Arg729Gly |
| 19 | NF1 | 17 | 29553581 | CCG | C | 0.063 | Frameshift | NM_000267.3 | c.2132_2133del | NP_000258.1 | p.Arg711ProfsTer4 |
| 20 | EZH2 | 7 | 1.49E+08 | G | T | 0.029 | Stop gained | NM_001203247.1 | c.251C>A | NP_001190176.1 | p.Ser84Ter |
| 21 | U2AF1 | 21 | 44524456 | G | A | 0.049 | Missense | NM_006758.2 | c.101C>T | NP_006749.1 | p.Ser34Phe |
| 21 | U2AF1 | 21 | 44524456 | G | T | 0.049 | Missense | NM_006758.2 | c.101C>A | NP_006749.1 | p.Ser34Tyr |
| 22 | IKZF3 | 17 | 37922471 | C | A | 0.099 | Stop gained | NM_012481.4 | c.1102G>T | NP_036613.2 | p.Glu368Ter |
| 23 | BCOR | X | 39921447 | AC | A | 0.028 | Frameshift | NM_001123385.1 | c.4372del | NP_001116857.1p.Val1458SerfsTer26 | p.Val1458SerfsTer26 |
| 23 | KMT2A | 11 | 1.18E+08 | G | T | 0.096 | Stop gained | NM_005933.3 | c.7987G>T | NP_005924.2 | p.Gly2663Ter |
| 23 | SETDB1 | 1 | 1.51E+08 | G | T | 0.041 | Stop gained | NM_001145415.1 | c.277G>T | NP_001138887.1 | p.Glu93Ter |
| 24 | CREBBP | 16 | 3786183 | C | A | 0.053 | Stop gained | NM_004380.2 | NM_004380.2:c.4582G>T | NP_004371.2 | p.Glu1528Ter |
| 24 | DNMT3A | 2 | 25467023 | C | A | 0.13 | Splice donor | NM_022552.4 | c.1851+1G>T |  |  |
| 24 | JAK2 | 9 | 5073770 | G | T | 0.048 | Missense | NM_004972.3 | c.1849G>T | NP_004963.1 | p.Val617Phe |
| 25 | EP300 | 22 | 41554448 | C | A | 0.057 | Stop gained | NM_001429.3 | c.3534C>A | NP_001420.2 | p.Tyr1178Ter |
| 26 | PHIP | 6 | 79680536 | TG | T | 0.031 | Frameshift | NM_017934.5 | c.2958del | NP_060404.3 | p.Asn987IlefsTer18 |
| 27 | CUX1 | 7 | 1.02E+08 | G | T | 0.046 | Stop gained | NM_181552.3 | c.3691G>T | NP_853530.2 | p.Glu1231Ter |
| 28 | PDS5B | 13 | 33338693 | GA | G | 0.037 | Frameshift | NM_015032.3 | c.3592del | NP_055847.1 | p.Ser1198ValfsTer10 |
| 28 | U2AF1 | 21 | 44524456 | G | A | 0.056 | Missense | NM_006758.2 | c.101C>T | NP_006749.1 | p.Ser34Phe |
| 28 | U2AF1 | 21 | 44524456 | G | T | 0.056 | Missense | NM_006758.2 | c.101C>A | NP_006749.1 | p.Ser34Tyr |
| 29 | STAG1 | 3 | 1.36E+08 | TC | T | 0.07 | Frameshift | NM_005862.2 | c.2896del | NP_005853.2 | p.Asp966ThrfsTer17 |
| 30 | TET2 | 4 | 1.06E+08 | T | TCC | 0.071 | Frameshift | NM_001127208.2 | c.1380_1381dup | NP_001120680.1 | p.Gln461ProfsTer26 |
| 31 | SF1 | 11 | 64543969 | C | A | 0.059 | Splice donor | NM_004630.3 | c.160+1G>T |  |  |
| 32 | SETD2 | 3 | 47164400 | TAC | T | 0.028 | Frameshift | NM_014159.6 | c.1724_1725del | NP_054878.5 | p.Cys575TyrfsTer5 |
| 33 | TP53 | 17 | 7573943 | T | TC | 0.166 | Frameshift | NM_001126112.2 | c.1083dup | NP_001119584.1 | p.Ser362GlufsTer20 |
| 34 | IKZF3 | 17 | 37985716 | G | T | 0.078 | Stop gained | NM_012481.4 | c.87C>A | NP_036613.2 | p.Tyr29Ter |
| 35 | RAD21 | 8 | 1.18E+08 | A | T | 0.078 | Stop gained | NM_006265.2 | c.633T>A | NP_006256.1 | p.Tyr211Ter |
| 36 | PDS5B | 13 | 33338693 | GA | G | 0.028 | Frameshift | NM_015032.3 | c.3592del | NP_055847.1 | p.Ser1198ValfsTer10 |
| 37 | EED | 11 | 85967532 | CTA | C | 0.032 | Frameshift | NM_003797.3 | c.533_534del | NP_003788.2 | p.Ile178AsnfsTer17 |
| 38 | SETD2 | 3 | 47162321 | TAG | T | 0.033 | Frameshift | NM_014159.6 | c.3803_3804del | NP_054878.5 | p.Ser1268TyrfsTer7 |
| 39 | SMC3 | 10 | 1.12E+08 | G | T | 0.046 | Stop gained & splice region | NM_005445.3 | c.721G>T | NP_005436.1 | p.Glu241Ter |
| 40 | ETV6 | 12 | 11905438 | TCG | T | 0.062 | Frameshift | NM_001987.4 | c.89_90del | NP_001978.1 | p.Ser30TyrfsTer35 |
| 41 | KMT2A | 11 | 1.18E+08 | A | AC | 0.069 | Frameshift | NM_005933.3 | c.2318dup | NP_005924.2 | p.Ser774ValfsTer12 |
| 42 | TET2 | 4 | 1.06E+08 | GC | G | 0.063 | Frameshift | NM_001127208.2 | c.2596del | NP_001120680.1 | p.Gln866AsnfsTer7 |
| 43 | DNMT3A | 2 | 25497919 | TCC | T | 0.037 | Frameshift & stop gained | NM_022552.4 | c.528_529del | NP_072046.2 | p.Trp176Ter |
| 44 | CBL | 11 | 1.19E+08 | G | A | 0.047 | Missense | NM_005188.3 | c.1259G>A | NP_005179.2 | p.Arg420Gln |
| 45 | BCORL1 | X | 1.29E+08 | T | TC | 0.078 | Frameshift | NM_021946.4 | c.5042dup | NP_068765.3 | p.Gly1682ArgfsTer4 |
| 45 | KMT2A | 11 | 1.18E+08 | A | AC | 0.093 | Frameshift | NM_005933.3 | c.2318dup | NP_005924.2 | p.Ser774ValfsTer12 |
| 45 | PDS5B | 13 | 33306277 | A | AC | 0.062 | Frameshift | NM_015032.3 | c.2170dup | NP_055847.1 | p.Arg724ProfsTer16 |
| 45 | SF3A1 | 22 | 30737857 | T | TG | 0.094 | Frameshift | NM_005877.4 | c.894dup | NP_005868.1 | p.Thr299HisfsTer17 |
| 45 | TP53 | 17 | 7573943 | T | TC | 0.112 | Frameshift | NM_001126112.2 | c.1083dup | NP_001119584.1 | p.Ser362GlufsTer20 |
| 46 | CUX1 | 7 | 1.02E+08 | G | T | 0.043 | Stop gained | NM_181552.3 | c.2530G>T | NP_853530.2 | p.Glu844Ter |

| **Supplemental Table 3: Rates of Acute Rejection during the first year after transplant** | | | |
| --- | --- | --- | --- |
|  | **Overall**  **(n = 269)** | **CHIP variant present**  **(n = 42)** | **CHIP variant absent**  **(n = 227)** |
| A Grade Rejection Score**^*^** | 0.33 (0, 2) | 0.32 (0, 1) | 0.33 (0, 2) |
| Episodes of acute rejection **^**^**  A1  A2 or higher | 142 (52.8%)  122 (45.4%) | 24 (57.1%)  18 (42.9%) | 118 (52.0%)  104 (45.8%) |
| Continuous variables presented as median (min, max); categorical variables presented as frequency (proportion)  * Sum of ISHLT grade A scores divided by the number of gradable transbronchial biopsies  **^**^**Indicates number of participants with at least one A1 or A2 or higher biopsies. Percentages will not sum to 1. | | | |

| **Supplemental Table 4: Breakdown of CLAD-free survival events** | | | |
| --- | --- | --- | --- |
|  | **No CHIP variant (n=234)** | **CHIP variant identified (n=45)** | **Overall (n=279)** |
| **Developed CLAD** | 98 (41.9%) | 21 (46.7%) | 119 (42.7%) |
| **Died without CLAD** | 96 (41.0%) | 17 (37.8%) | 113 (40.5%) |
| **Re-transplant without CLAD** | 1 (0.4%) | 0 (0%) | 1 (0.4%) |
| **CLAD/Died/Re-transplant** |  |  |  |
| No | 39 (16.7%) | 7 (15.6%) | 46 (16.5%) |
| Yes | 195 (83.3%) | 38 (84.4%) | 233 (83.5%) |
| **Time To Event/Censor (days)** |  |  |  |
| Mean (SD) | 1720 (1380) | 1300 (1360) | 1650 (1380) |

| **Supplemental Table 5: Association between *DNMT3A* and *TET2* variants and death or CLAD** | | |
| --- | --- | --- |
| *Variable* | *HR (95% CI)* | *p-value* |
| CHIP due to *TET2* or *DNMT3A* variant vs No CHIP | 1.28 (0.68 – 2.41) | 0.44 |
| CHIP due to variant in gene other than *TET2/DNMT3A* vs No CHIP | 1.24 (0.823-1.857) | 0.31 |
| Age at transplant | 1.02 (1.00 – 1.03) | 0.02 |
| Female | 1.05 (0.80 – 1.39) | 0.72 |
| Grade 3 PGD at 72 hours | 1.57 (1.06 – 2.32) | 0.02 |
| Single lung transplant |  |  |
| Before 1200 days | 0.86 (0.56 – 1.31) | 0.48 |
| After 1200 days | 2.38 (1.42 – 3.99) | <0.001 |

| **Supplemental Table 6: Association between CLAD-free survival and CHIP in patients with Pulmonary Fibrosis adjusting for telomere-related gene variant (N=173)** | | |
| --- | --- | --- |
| *Variable* | *HR (95% CI)* | *p-value* |
| CHIP variant |  | <0.001* |
| 1 CHIP variant | 1.07 (0.65 – 1.80) |  |
| > 1 CHIP variant | 4.08 (1.95 – 8.55) |  |
| Telomere-related gene variant** | 1.84 (1.15 – 2.96) | 0.01 |
| Age at transplant | 1.02 (1.00 – 1.05) | 0.06 |
| Female | 1.24 (0.86 – 1.79) | 0.25 |
| Grade 3 PGD | 1.27 (0.78 – 2.09) | 0.34 |
| Single lung transplant |  |  |
| Before 1200 days | 0.72 (0.44 – 1.18) | 0.19 |
| After 1200 days | 1.40 (0.80 – 2.46) | 0.23 |
| *Represents the p-value for any difference by CHIP variant.  **Defined as qualifying variant in *TERT, RTEL1,* or *PARN*, as previously described (3) | | |

**Supplemental Figure 1**: The prevalence of CHIP variants increases with age at transplant in both males and females


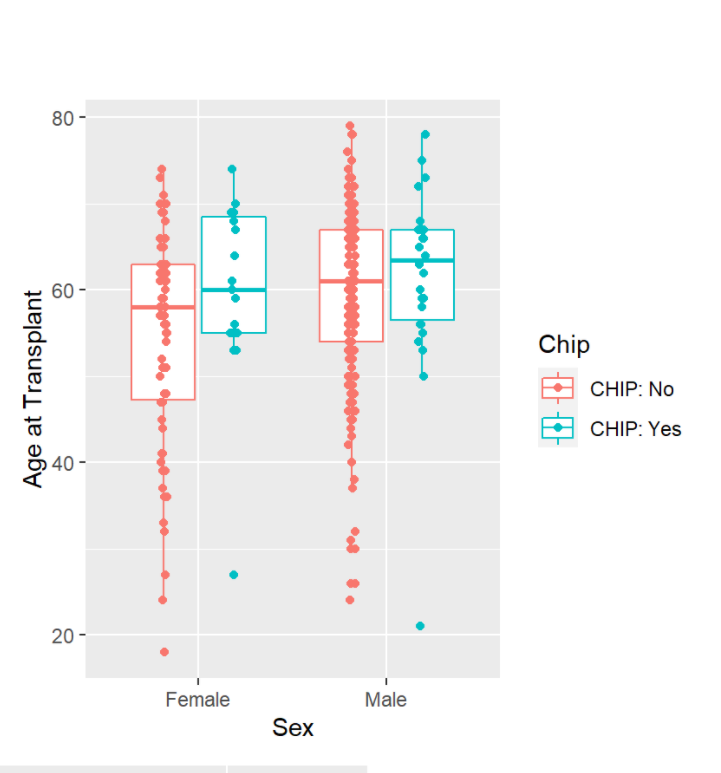


**Supplemental Figure 2:** Mean Sequencing Depth among patients with and without CHIP variants


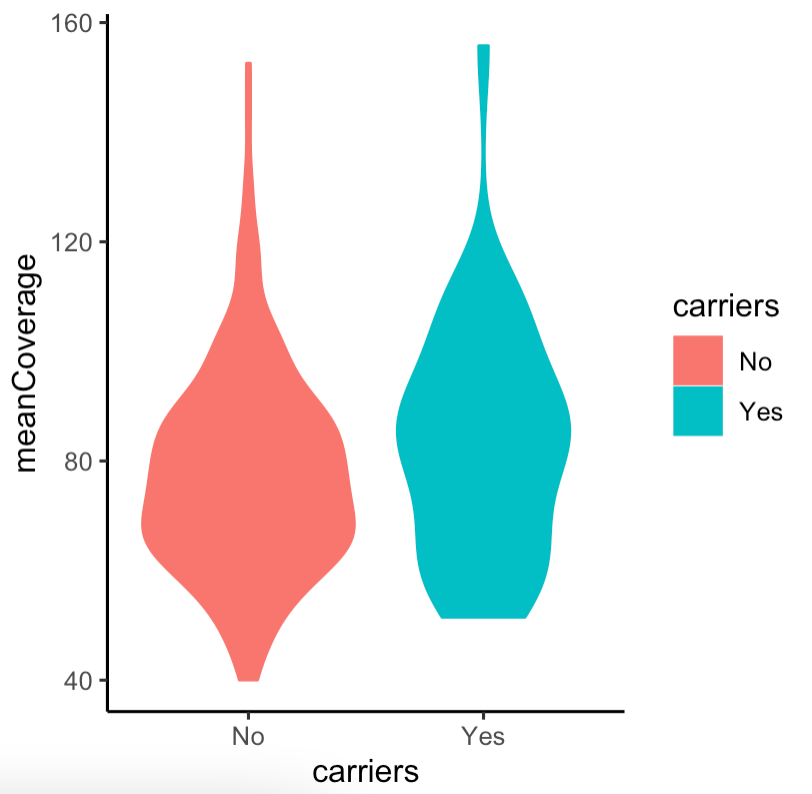


CHIP absent

CHIP present

CHIP

Absent

Present

Mean Sequencing Depth

**Supplemental Figure 3:** Median Acute Rejection (AR) Score in the first post-transplant year in individuals with CHIP vs without CHIP.


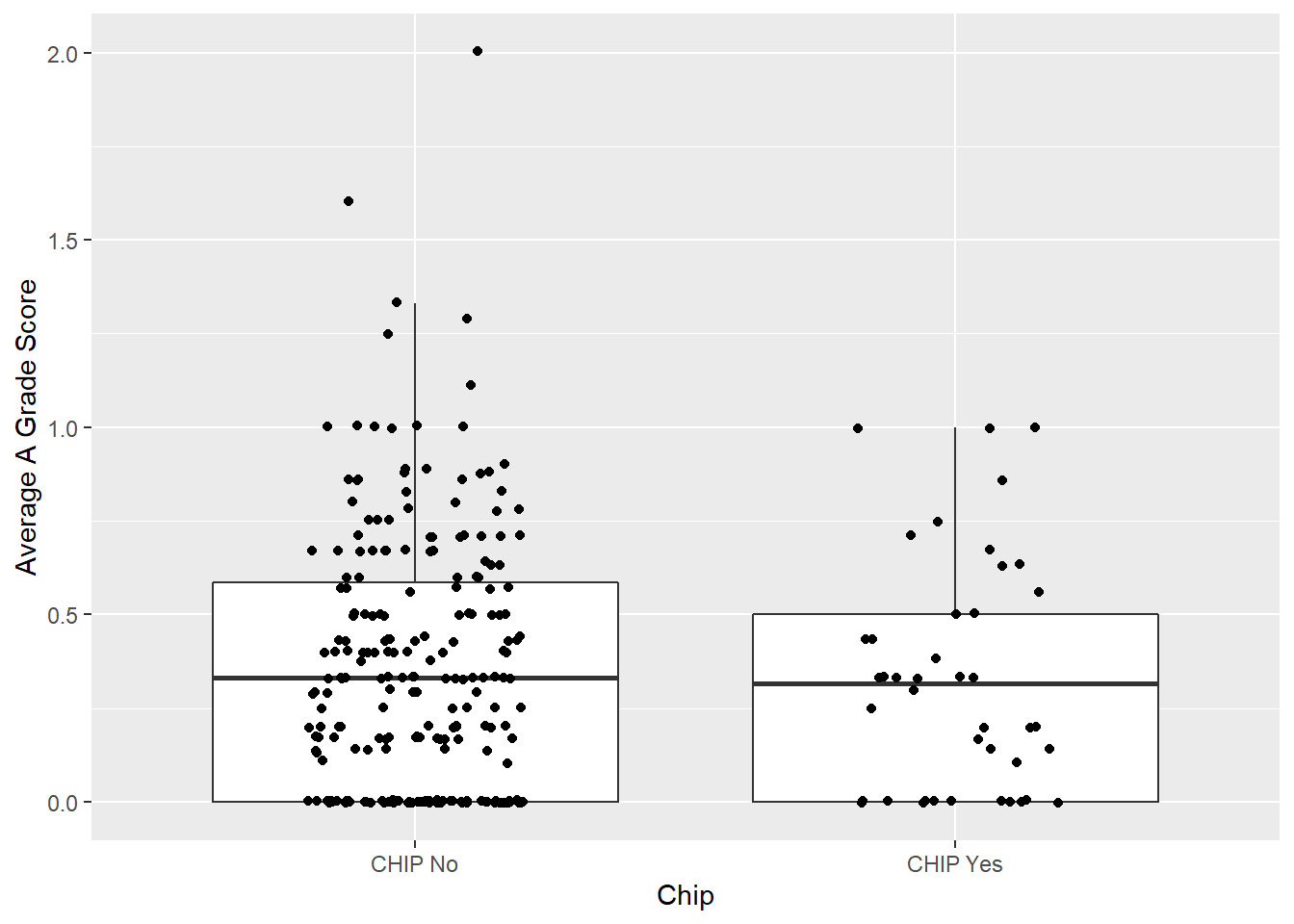


**Supplemental Figure 4:** Kaplan Meier curve for time to development of CLAD or death after lung transplant stratified by the presence CHIP variants and telomere related gene variants


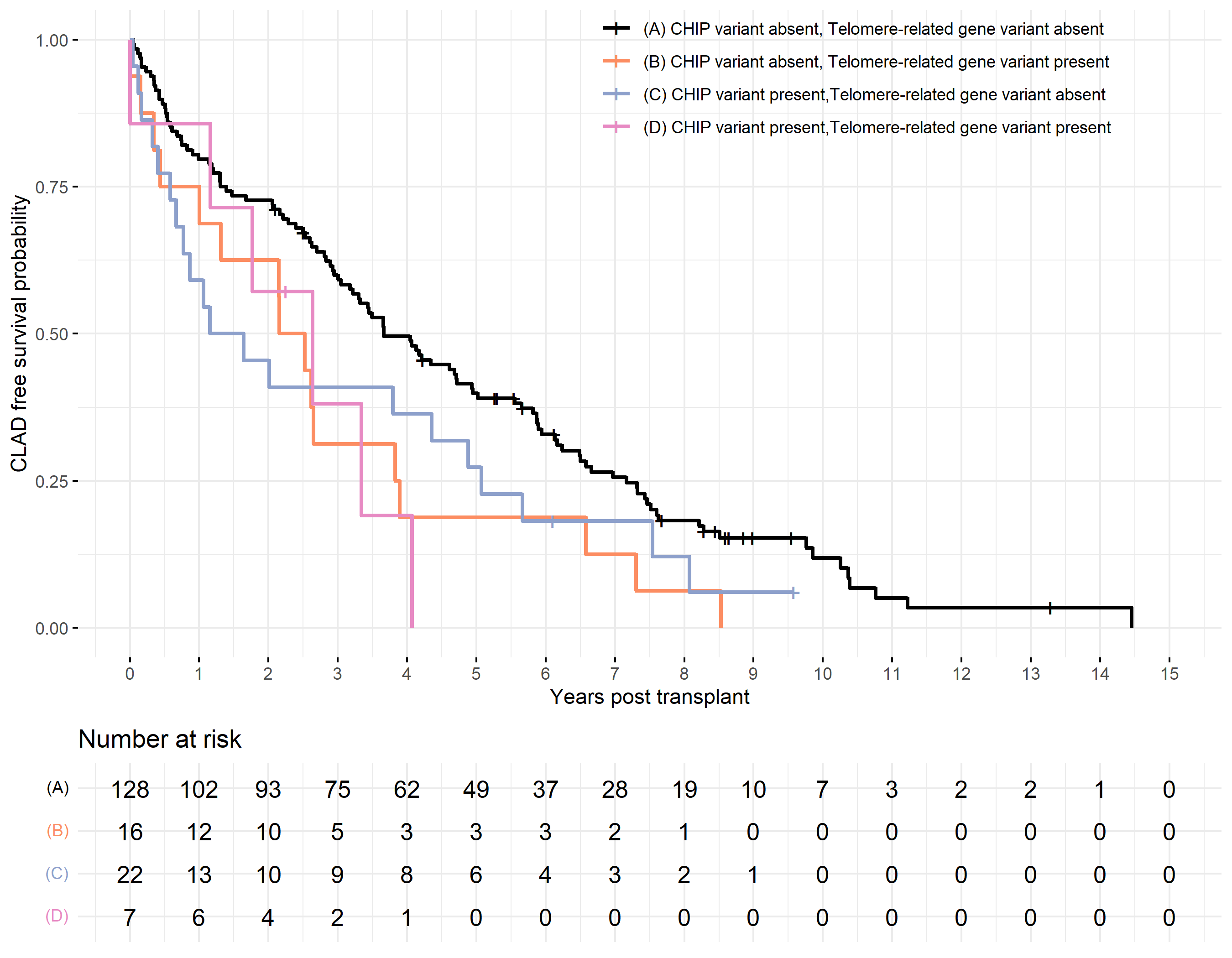


**Supplemental Figure 5:** Forrest plot depicting hazard ratios for CLAD-free survival among 179 patients with pulmonary fibrosis who were evaluated for both telomere-related gene variants and CHIP variants.


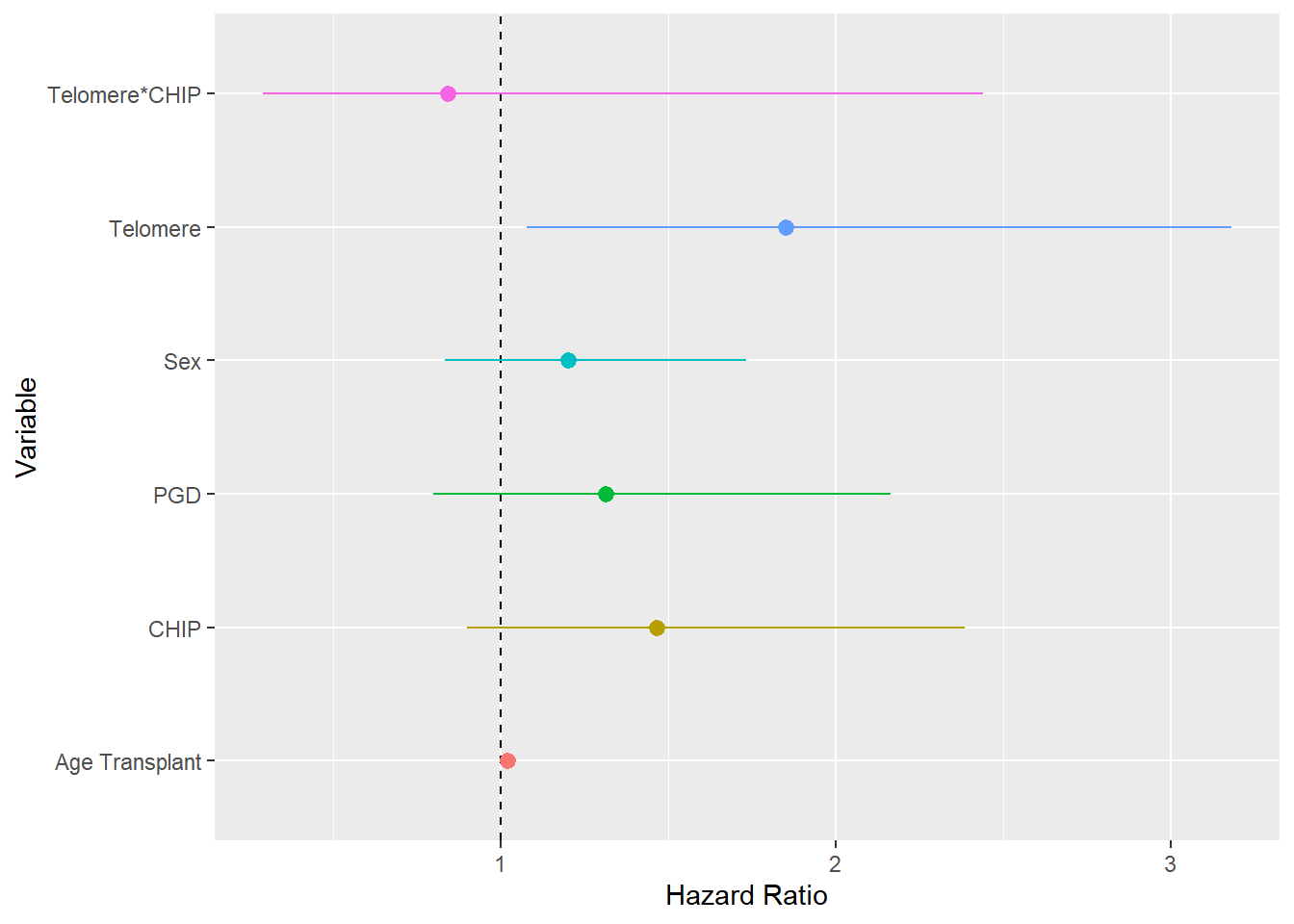


1. Petrovski S, Todd JL, Durheim MT, et al. An Exome Sequencing Study to Assess the Role of Rare Genetic Variation in Pulmonary Fibrosis. *Am J Respir Crit Care Med*. Jul 1 2017;196(1):82-93. doi:10.1164/rccm.201610-2088OC
